# Expression of Chemoresistance-Associated ABC Proteins in Hepatobiliary, Pancreatic and Gastrointestinal Cancers

**DOI:** 10.3390/cancers14143524

**Published:** 2022-07-20

**Authors:** Jose J. G. Marin, Maria J. Monte, Rocio I. R. Macias, Marta R. Romero, Elisa Herraez, Maitane Asensio, Sara Ortiz-Rivero, Candela Cives-Losada, Silvia Di Giacomo, Javier Gonzalez-Gallego, Jose L. Mauriz, Thomas Efferth, Oscar Briz

**Affiliations:** 1 Experimental Hepatology and Drug Targeting (HEVEPHARM) Group, University of Salamanca, IBSAL, 37007 Salamanca, Spain; mjmonte@usal.es (M.J.M.); rociorm@usal.es (R.I.R.M.); marta.rodriguez@usal.es (M.R.R.); elisah@usal.es (E.H.); masensio002@usal.es (M.A.); saraortizriv@usal.es (S.O.-R.); candelacives@usal.es (C.C.-L.); 2 Centro de Investigación Biomédica en Red de Enfermedades Hepáticas y Digestivas (CIBERehd), Carlos III National Institute of Health, 28029 Madrid, Spain; jgonga@unileon.es (J.G.-G.); jl.mauriz@unileon.es (J.L.M.); 3 Department of Physiology and Pharmacology “Vittorio Erspamer”, Sapienza University of Rome, 00185 Rome, Italy; silvia.digiacomo@uniroma1.it; 4 Institute of Biomedicine (IBIOMED), University of León, Campus of Vegazana s/n, 24071 Leon, Spain; 5 Department of Pharmaceutical Biology, Institute of Pharmaceutical and Biomedical Sciences, Johannes Gutenberg University, Staudinger Weg 5, 55128 Mainz, Germany; efferth@uni-mainz.de

**Keywords:** ATP-binding cassette protein, anticancer drug, drug refractoriness, multidrug resistance, transport

## Abstract

**Simple Summary:**

One-third of the approximately 10 million deaths yearly caused by cancer worldwide are due to hepatobiliary, pancreatic, and gastrointestinal tumors. One primary reason for this high mortality is the lack of response of these cancers to pharmacological treatment. More than 100 genes have been identified as responsible for seven mechanisms of chemoresistance, but only a few of them play a critical role. These include ABC proteins (mainly MDR1, MRP1-6, and BCRP), whose expression pattern greatly determines the individual sensitivity of each tumor to pharmacotherapy.

**Abstract:**

Hepatobiliary, pancreatic, and gastrointestinal cancers account for 36% of the ten million deaths caused by cancer worldwide every year. The two main reasons for this high mortality are their late diagnosis and their high refractoriness to pharmacological treatments, regardless of whether these are based on classical chemotherapeutic agents, targeted drugs, or newer immunomodulators. Mechanisms of chemoresistance (MOC) defining the multidrug resistance (MDR) phenotype of each tumor depend on the synergic function of proteins encoded by more than one hundred genes classified into seven groups (MOC1-7). Among them, the efflux of active agents from cancer cells across the plasma membrane caused by members of the superfamily of ATP-binding cassette (ABC) proteins (MOC-1b) plays a crucial role in determining tumor MDR. Although seven families of human ABC proteins are known, only a few pumps (mainly MDR1, MRP1-6, and BCRP) have been associated with reducing drug content and hence inducing chemoresistance in hepatobiliary, pancreatic, and gastrointestinal cancer cells. The present descriptive review, which compiles the updated information on the expression of these ABC proteins, will be helpful because there is still some confusion on the actual relevance of these pumps in response to pharmacological regimens currently used in treating these cancers. Moreover, we aim to define the MOC pattern on a tumor-by-tumor basis, even in a dynamic way, because it can vary during tumor progression and in response to chemotherapy. This information is indispensable for developing novel strategies for sensitization.

## 1. Introduction

**Figure 1:**
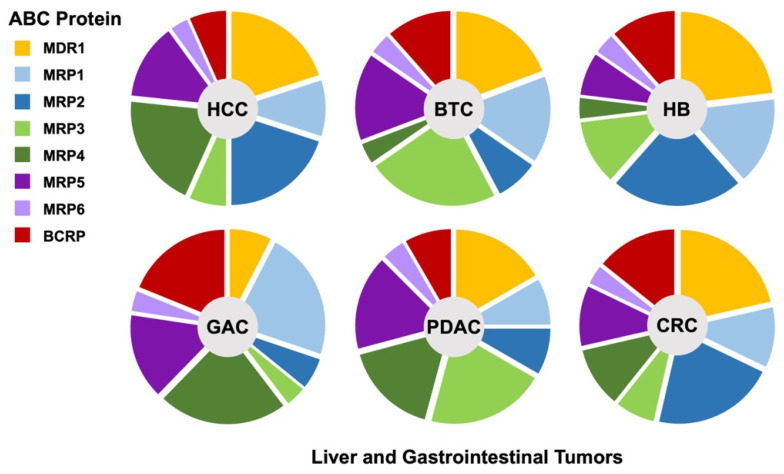
Expression pattern of ABC proteins involved in chemoresistance of liver and gastrointestinal cancers. HCC, hepatocellular carcinoma; BTC, biliary tract cancer; HB, hepatoblastoma; GAC, gastric adenocarcinoma; PDAC, pancreatic ductal adenocarcinoma; CRC, colorectal carcinoma.

Hepatobiliary, pancreatic and gastrointestinal tumors account for approximately 36% of deaths due to cancer worldwide. One of the main reasons for this high mortality is their late diagnosis and high refractoriness to pharmacological treatments. In this way, most of these tumors are detected when curative approaches, such as surgical resection, are no longer advisable. The second cause of poor outcomes is their high refractoriness to pharmacological treatments, i.e., classical chemotherapy, targeted drugs, and newer immunomodulators, which may constitute the only therapeutical option for these patients with advanced cancers. More than 100 genes involved in the lack of response to drug therapy have been identified and classified into seven groups of mechanisms of chemoresistance (MOC) [1,2,3]. Among them, the efflux of active agents from cancer cells across the plasma membrane through members of the superfamily of ATP-binding cassette (ABC) proteins plays a crucial role in drug resistance mechanisms categorized as MOC-1b. Although there are seven families of ABC proteins in humans (from ABCA to ABCG), only members of the ABCB, ABCC, and ABCG families have been clearly associated with anticancer drug export (Table 1) [4]. Thus, the first member related to chemoresistance was named multidrug resistance protein 1 (MDR1), also known as P-glycoprotein or P-gp (gene symbol *ABCB1*). This pump can transport a large variety of anticancer drugs and is highly expressed in many tumors of the digestive system (Table 1). Several members of the ABCC family, known as multidrug resistance-associated proteins (MRPs), have also been associated with the lack of response to these tumors, namely MRP1 (*ABCC1*), MRP2 (*ABCC2*), MRP3 (*ABCC3*), MRP4 (*ABCC4*), MRP5 (*ABCC5*), and MRP6 (*ABCC6*). Although there is some overlap regarding substrate-specificity, there are also significant differences in this respect (Table 1). Moreover, their expression in these tumors shows characteristic patterns (Figure 1). Finally, there is a member of the ABCG family, the breast cancer resistance protein or BCRP (*ABCG2*), which plays a relevant role in the transport of anticancer drugs and hence in the chemoresistance of digestive cancers (Table 1). MDR1 and MRP1-6 are full transporters, whereas *ABCG2* is a half-transporter that requires dimerization to become functionally active. In addition to their basal expression in naïve tumors, a common feature in resistance development is their upregulation in response to treatment [4]. Nevertheless, there is also the possibility of manipulating their expression [5] and function [6]. The present descriptive review compiles updated information on the expression of ABC proteins involved in the multidrug resistance (MDR) phenotype of liver and gastrointestinal cancers.

## 2. Hepatocellular Carcinoma

Hepatocellular carcinoma (HCC), which derives from hepatocytes, is the most frequent primary liver cancer, causing more than 800,000 deaths per year worldwide, i.e., more than 8% of deaths due to cancer (Global Cancer Observatory; http://gco.iarc.fr accessed on 1 June 2022). Systemic drug therapy is the only therapeutic option for most patients with unresectable advanced HCC. However, one important feature accounting for the high mortality of HCC is its marked drug refractoriness mainly to classical chemotherapy, whose current use is limited to locoregional therapies, such as transarterial chemoembolization (TACE) with doxorubicin, cisplatin, or 5-fluorouracil (5-FU) in a small group of patients. Moreover, alternative treatments based on tyrosine kinase inhibitors (TKIs), such as sorafenib, lenvatinib, regorafenib, and cabozantinib, or even immunotherapy, provide very modest beneficial effects [7]. This is in part due to drug efflux from cancer cells through ABC proteins, which play a crucial role in the sensitivity of pharmacotherapy in HCC (Table 2).

### 2.1. MDR1 in Hepatocellular Carcinoma

MDR1, or P-glycoprotein, is expressed in the canalicular membrane of hepatocytes [23], where it exports amphiphilic cations, endogenous steroids, and xenobiotics into bile. MDR1 expression is highly variable among HCC tumors. Marked upregulation of MDR1 has been reported in some cases [24], whereas, in many others, MDR1 abundance (both mRNA and protein) is lower in the tumor than in adjacent liver tissue [25]. HCC-derived cell lines also show marked variability in MDR1 expression. Most of these cell lines express MDR1 only at low levels, whereas in a few of them, such as HuH-7 and Hep3B, this pump is highly expressed [26]. In the clinical setting, MDR1 expression is associated with short overall survival (OS) of HCC patients [8]. There is an inverse relationship between MDR1 expression levels in the tumor and the response to systemic chemotherapy of advanced HCC [25]. This is consistent with the fact that MDR1 is considered an important factor in determining the response of HCC to sorafenib [9]. Other TKIs approved by the FDA for the treatment of HCC, such as regorafenib and lenvatinib, are also MDR1 substrates [27,28]. Elevated MDR1 expression in the tumor has been associated with lower accumulation of doxorubicin and worse prognosis in patients treated with this drug, making doxorubicin scarcely effective against HCC [10].

### 2.2. MRP1 in Hepatocellular Carcinoma

Under basal conditions, healthy hepatocytes express MRP1 at very low, almost undetectable levels in the basolateral membrane [29]. However, in HCC, variable MRP1 expression has been reported. Some authors have found overexpression of this transporter in tumors compared to the surrounding tissue [30], while no difference [31] or even undetectable MRP1 protein levels [32] have been reported by others. Despite the well-known ability of MRP1 to export many antitumor drugs [33], there is scarce information on the role of this pump in the overall refractoriness to TKIs used in the treatment of HCC. In this sense, sorafenib seems to induce MRP1 expression in HCC-derived cells, presumably through ubiquitin peptidase 22 (UPS22) upregulation [13]. Consistently, positive staining of UPS22 and MRP1, assessed by immunohistochemistry, correlated with each other in sorafenib-resistant tumors, but not in sorafenib-sensitive ones [13].

### 2.3. MRP2 in Hepatocellular Carcinoma

MRP2 is highly expressed in the canalicular membrane of hepatocytes, where it plays an essential role in liver detoxification and chemoprotection processes [34]. MRP2 is also highly expressed in HCC [35] and, therefore, could contribute to its MDR phenotype. Indeed, MRP2 modulates the intracellular accumulation of sorafenib [14] due to the extrusion of the drug itself or its metabolite sorafenib glucuronide [36]. Moreover, MRP2 can also export regorafenib [37], cabozantinib [38], and probably lenvatinib [39].

### 2.4. MRP3 in Hepatocellular Carcinoma

Although with high individual variability, MRP3 expression shows a trend of decreasing levels in HCC compared with adjacent liver tissue [27,33,34]. Thus, in a recent study, only 18.8% of HCC (15 out of 80) showed positive staining for MRP3 by immunohistochemistry [40], suggesting a minor role of this ABC transporter in HCC chemoresistance. Nevertheless, a relationship between MRP3-mediated efflux and sensitivity to sorafenib in HCC cells has been reported [15].

### 2.5. MRP4 in Hepatocellular Carcinoma

In healthy hepatocytes, MRP4 is poorly expressed in their basolateral membrane [17]. In contrast, this pump is upregulated in cholestatic liver diseases, favoring the regurgitation of cholephilic compounds toward the sinusoidal blood. Similarly, MRP4 is also upregulated in HCC [41]. MRP4 expression is also high in cell lines of hepatic origins, such as HuH-7 [42] and HepaRG [43]. MRP4 mediates the extrusion of xenobiotics together with reduced glutathione (GSH) [17]. Studies performed on PLC/PRF5 cells exposed to sorafenib indicated that MRP4 is not involved in HCC refractoriness to this TKI [15]. Although cisplatin is not a known substrate of MRP4, in vitro studies have shown that cisplatin-resistant HCC cells exhibit MRP4 upregulation [16], which can be involved in developing cross-resistance.

### 2.6. MRP5 in Hepatocellular Carcinoma

MRP5 is expressed in the basal plasma membrane of many epithelial cells, including hepatocytes [44]. Some authors have described an increased MRP5 expression in untreated HCC compared to adjacent non-tumor liver tissue [18, 41]. In contrast, other studies have failed to detect MRP5 in either tumor or healthy liver parenchyma [45]. MRP5 is unambiguously detected (both mRNA and protein) in HCC-derived cell lines such as HuH-7, Hep3B, and HLF [39,40,44]. Some reports have also shown that in cisplatin- and doxorubicin-resistant HCC cells, MRP5 expression is higher than in wild-type cells [16,19]. Typical endogenous substrates of MRP5 are cyclic nucleotides, such as cAMP and cGMP [46]. In addition, this transporter can mediate the export of 5-FU, thus reducing the sensitivity of HCC cells to this drug [47]. However, so far, no clinical data on the relevance of MRP5 in the lack of response of HCC to chemotherapy or TKIs are available.

### 2.7. BCRP in Hepatocellular Carcinoma

BCRP is expressed in the apical membrane of hepatocytes [48] and in a very heterogeneous way in HCC-derived cells. Although some studies have reported non-significant upregulation of *ABCG2* mRNA in HCC [41], in general, BCRP expression is higher in tumor tissue than in adjacent non-tumor liver tissue and healthy hepatocytes [49,50]. Moreover, *ABCG2* mRNA levels in HCC-derived cells are higher in undifferentiated than in differentiated cell lines [49]. *ABCG2* expression is typically very high in the so-called side-population (SP) of stem cells, whose chemoresistance is stronger than those without stemness characteristics [51]. Thus, BCRP is a potential marker for liver cancer stem cells (LCSC) [52,53]. Interestingly, BCRP expression is closely related to the initiation, proliferation, metastasis, and chemoresistance of HCC. Increased BCRP expression has been correlated with reduced OS in HCC of elderly patients [50]. Surprisingly, other studies have reported that HCC patients with low BCRP expression have a significantly shorter OS and recurrence-free survival time after treatment with epirubicin or cisplatin, alone or in combination with 5-FU [54]. *ABCG2* expression, which can be modulated by AKT signaling, can significantly influence the efflux from HCC cells of drugs such as doxorubicin, therefore hindering their efficacy [54]. In addition, the development in HCC cells of an LCSC phenotype, in which *ABCG2* upregulation is prominent, is associated with malignant traits, such as increased proliferation, migration, and invasion [53]. It is noteworthy that these features can be diminished by *ABCG2* downregulation [52]. BCRP is one of the most relevant ABC proteins in determining the response of HCC patients to sorafenib [9]. BCRP plays a dominant role in sorafenib efflux [55], and it has been proposed as a predictor of HCC response to this drug [21]. In addition, HCC patients who carry the genetic variants rs2231137 (c.34G>A, p.Val12Met) and rs2231142 (c.421C>G/A, p.Gln141Glu/Lys) of *ABCG2* and rs1045642 (c.3645T>G, p.Ile1215Met) of *ABCB1* have lower plasma levels of sorafenib and show better progression-free survival [11]. However, the presence of these variants does not affect the pharmacokinetics of lenvatinib.

### 2.8. Other ABC Pumps in Hepatocellular Carcinoma

The involvement of other ABC proteins, such as ABCA2, ABCA3, ABCA6, ABCA8, MRP6, and MRP7 (*ABCC10*), in the development of the MDR phenotype in HCC, is considered scarcely relevant [56]. ABCB5 and ABCF1 are LCSC markers. Cell subpopulations with stemness properties, obtained from biopsies of HCC patients, express higher levels of ABCB5 than the surrounding non-tumor tissue [57]. Using Hep3B cells, the overexpression of ABCB5 has been related to the development of doxorubicin resistance [12]. ABCF1 is a hepatic oncofetal protein upregulated in HCC, which has recently been reported to promote resistance to cisplatin and doxorubicin using both in vitro and in vivo models [22].

## 3. Biliary Tract Cancer

Biliary tract cancer (BTC) refers to a group of rare and aggressive malignancies arising in the biliary tree that comprises cholangiocarcinoma (CCA), gallbladder cancer (GBC), and ampullary cancer. Options of systemic chemotherapy for treating patients with advanced BTC are similar in all cases, including in the first-line combinations of gemcitabine and cisplatin or oxaliplatin plus 5-FU (FOLFOX) or capecitabine (CAPOX). In the second-line setting, alternatives, such as FOLFIRINOX (5-FU, irinotecan, and oxaliplatin) and etoposide toniribate, have been assayed. Finally, novel targeted drugs and immunotherapy offer hope for the future of these patients, although no efficient therapy currently exists [58]. As part of the BTC “transportome”, defined as the set of transporters expressed at a given moment in the tumor, which is an essential element for defining its MDR phenotype, ABC proteins play a crucial role [59] (Table 3).

### 3.1. MDR1 in Biliary Tract Cancer

The presence of MDR1 in the apical plasma membrane of the bile duct and gallbladder epithelial cells suggests that this export pump plays a physiological role in preventing the accumulation of endogenous and xenobiotic compounds secreted into bile in cells of the biliary tree by exporting them back to bile [23,68]. MDR1 expression levels are maintained in CCA and GBC [41,68] but are usually decreased in poorly differentiated tumors [69]. Accordingly, MDR1 is considered a contributing factor to the MDR phenotype of these tumors [70]. In fact, in patients with GBC, high MDR1 levels have been associated with increased resistance to gemcitabine [60].

### 3.2. MRP1 in Biliary Tract Cancer

Although MRP1 is not detected in normal cholangiocytes and gallbladder epithelium [71], its expression has been found elevated in some cases of intrahepatic CCA (iCCA) [63]. MRP1 has been shown to confer resistance to gemcitabine in CCA-derived cells [61]. Several studies have also associated MRP1 downregulation with increased sensitivity to 5-FU of CCA-derived cells in vitro [62,72]. Moreover, in patients with iCCA or GBC who received adjuvant chemotherapy, their poor prognosis was associated with high MRP1 mRNA levels in these tumors [63,64].

### 3.3. MRP2 in Biliary Tract Cancer

MRP2 is expressed in the apical membrane of cholangiocytes and gallbladder epithelial cells, where it plays a role in the barrier for anionic metabolites present in bile [71]. In a study carried out in 2008, it was suggested that MRP2 is not relevant in BTC chemoresistance because this protein was detected by immunohistochemistry in only 4 out of 14 GBC analyzed and was not detectable in any CCA [73]. However, subsequent studies, including a more significant number of cases, suggested the opposite conclusion because mRNA expression was found elevated in 28 out of 55 iCCA [63], and reactivity by immunohistochemistry was observed in most (96.3%) of the 56 CCA analyzed. A higher expression was seen in well or moderately differentiated tumors [40]. Moreover, MRP2 expression was found in half (53.1%) of the 143 GBC samples analyzed in another study [74]. Although marked MRP2 expression has not been found in human GBC cell lines [66,75], this is enhanced by incubation with cisplatin [76]. Moreover, MRP2 contributes to resistance to cisplatin, oxaliplatin, and gemcitabine in other types of tumors [77,78,79], which justifies the need to look further into its role in BTC resistance.

### 3.4. MRP3 in Biliary Tract Cancer

Under physiological conditions, MRP3 is expressed in the basolateral membrane of cholangiocytes [80] and gallbladder epithelial cells [71]. MRP3 is also markedly expressed in BTC. In a study carried out in 2008 [73], positive staining by immunohistochemistry was found in 13 of 14 GBC and 4 of 7 CCA. More recently, other studies have reported intense MRP3 expression in 44.5% of 56 CCAs [40] and in 74.5% of 59 GBCs, which was higher in more differentiated tumors [81]. MRP3 expression is also elevated in human CCA-derived cells [75]. Moreover, it has been demonstrated that MRP3 plays an important role in CCA chemoresistance, and, accordingly, it has been proposed as a target for chemosensitization [65].

### 3.5. MRP4, MRP5, and MRP6 in Biliary Tract Cancer

Although MRP4 can transport nitrogenous bases and nucleoside derivatives used against BTC, such as 5-FU and gemcitabine, its low expression in healthy biliary epithelium [82] and, although somewhat higher, in CCA [41] suggests that this pump has a minor role in BTC chemoresistance.

Tissue mRNA analyses revealed that MRP5 is ubiquitously expressed. High levels of this export pump able to transport derivatives of nitrogenous base and nucleosides have been found in CCA [41] and CCA-derived cell lines [66]. The exposure of HuCCT1 and KMBC cells, from iCCA and extrahepatic CCA (eCCA), respectively, to gemcitabine enhanced MRP5 expression. In contrast, MRP5 knockdown significantly increased gemcitabine cytotoxicity in KMBC cells [66]. However, an association between MRP5 expression and drug response in BTC patients has not been described yet.

The liver is one of the tissues with the highest expression levels of MRP6. Nevertheless, it is not clear whether this pump is expressed in cholangiocytes. Moreover, MRP6 has not been detected in gallbladder epithelium [83]. In contrast, HuCCT1 cells, derived from iCCA, show high mRNA and protein MRP6 levels, while no expression was detected in KMBC cells derived from eCCA. Gemcitabine upregulates MRP6 in HuCCT1 cells, and its knockdown enhances the sensitivity to this drug [66].

### 3.6. BCRP in Biliary Tract Cancer

BCRP is expressed in the apical membrane of cholangiocytes [84] and gallbladder epithelial cells [85], suggesting a protective role against potentially toxic compounds present in bile. Immunohistochemical analysis of 41 GBC specimens showed apical staining in well-differentiated tumors, whereas in poorly differentiated tumors, part of the signal was localized intracellularly [85]. Increased resistance to 5-FU has been described in CCA patients with high expression of BCRP, MDR1, and MRP3 [67].

## 4. Hepatoblastoma

Hepatoblastoma (HB) is the most common liver cancer in children and is treated with surgery and adjuvant or neoadjuvant antitumor chemotherapy [86]. Standard first-line chemotherapy is based on cisplatin plus doxorubicin. Although the response is better than that of HCC, it is not infallible because about 20% of HB patients do not respond to treatment and have a poor prognosis [87]. Other platinum and anthracycline derivatives, as well as other drugs such as etoposide, TKIs, *Vinca* alkaloids, 5-FU, irinotecan, and nitrogen mustards, are used as second-line treatment for refractory HB [88]. The bioavailability of some of these drugs is dependent on ABC pumps (Table 4).

### 4.1. MDR1 in Hepatoblastoma

MDR1 expression is usually high in HB [91] and can be further enhanced during drug treatment [91,92]. HB-derived cell lines exhibit a wide range of MDR1 expression [26], which is upregulated by incubating these cells with cisplatin or doxorubicin [92,93]. Similarly, mouse HB xenografts showed increased levels of MDR1 expression following treatment with doxorubicin [94] and cisplatin [95]. MDR1 confers resistance to anthracyclines in HB, as has been demonstrated in HepG2 cells overexpressing this pump [89]. Other drugs used in the second-line treatment of HB, such as etoposide, irinotecan, *Vinca* alkaloids, and sorafenib are also MDR1 substrates. Accordingly, upregulation of this ABC protein in HB may reduce its response to these drugs [88].

### 4.2. MRP1, MRP2, and MRP3 in Hepatoblastoma

MRP1 is highly expressed in HB compared to the surrounding liver tissue [88], as well as in HB-derived cell lines, such as HepG2 [41,96], HuH-6, and HepT1 [97]. However, there are discrepant results on the changes in MRP1 expression induced by the pharmacological treatment [98]. MRP1 plays an important role in anthracycline resistance [33] and is upregulated in HuH-6 cell spheroids, which decreases their sensitivity to doxorubicin [97]. Furthermore, the sensitivity to this drug was enhanced in HepG2 cells after silencing MRP1 [99].

MRP2 expression is high in HB [41] and is not changed in response to the treatment of these patients with cisplatin and doxorubicin [98]. HB-derived cell lines also express MRP2 [97]. Moreover, cisplatin-resistant HepG2 cells exhibited increased MRP2 levels [100]. Of note, MRP2 expression has been associated with the lack of response to cisplatin both in vitro [26] and in patients [90]. MRP2 could be involved in the resistance of HB to substrates of this pump, such as anthracyclines, irinotecan, sorafenib, etoposide, and *Vinca* alkaloids.

The levels of MRP3 expression are similar in healthy liver and HB tumor samples from untreated or standard chemotherapy-treated patients [98]. However, MRP3 has been found upregulated in HepG2 cells after exposure to cisplatin [41,101]. In experiments using cell models of MRP3 expression, this pump was able to transport sorafenib [15] and etoposide [102].

### 4.3. MRP4 and MRP5 in Hepatoblastoma

Although MRP4 could be involved in HB resistance to irinotecan, cyclophosphamide, 5-FU, and doxorubicin [88], its relevance is probably minor because MRP4 expression in healthy liver and HB is similarly low [41]. In contrast, HepG2 cells have high levels of MRP4 expression [103], which is further increased after the incubation with cisplatin [41].

MRP5 can determine tumor sensitivity to 5-FU [104] or anthracyclines [105]. However, its relevance in HB is uncertain. There are some discrepant results regarding MRP5 expression in HB. Previous determinations in a small number of patients reported no significant differences between HB compared to healthy liver tissue [41]. However, more recent reports suggested the existence of upregulated MRP5 expression in HB [88]. Moreover, the exposure of HepG2 cells to cisplatin did not further enhance their already elevated MRP5 expression [88].

### 4.4. BCRP in Hepatoblastoma

Although some studies have reported higher BCRP expression levels in HB than in healthy liver tissue, both at the mRNA and protein levels [41,98], the story is unclear because BCRP expression in the plasma membrane of HB cells has been reported to be low [98]. Another study has also found a decreased abundance of *ABCG2* mRNA in HB [88]. Moreover, BCRP expression was increased in the tumors after treating HB patients with cisplatin plus doxorubicin [98]. These findings were consistent with those obtained in HB-derived HuH-6 cells after short-term exposure to both drugs individually [88]. As anthracyclines, irinotecan, etoposide, sorafenib, and 5-FU are BCRP substrates, the expression of this ABC protein could affect the response of HB patients to these drugs [88].

### 4.5. Other ABC Pumps in Hepatoblastoma

The role of other ABC proteins in HB resistance is poorly understood. MRP6 and MRP7 are expressed in HB-derived cells and upregulated after cisplatin treatment [41,106]. These pumps can participate in resistance to etoposide, cisplatin, irinotecan, and anthracyclines [107,108].

## 5. Gastric Adenocarcinoma

Gastric adenocarcinoma (GAC) is a lethal type of cancer, accounting for approximately 770,000 deaths per year worldwide, which constitutes 7.7% of deaths due to cancer. The lack of availability of efficient pharmacological treatment for advanced GAC, either as neoadjuvant or adjuvant chemotherapy to surgical resection or radiotherapy [1,109] justifies the high mortality of this disease. As first-line treatment, the most used chemotherapeutic regimens include 5-FU, leucovorin, oxaliplatin, and perioperative docetaxel (FLOT) [110], and the combination of epirubicin, cisplatin, and 5-FU (ECF) [111]. However, other newer strategies such as immunotherapy or anti-VEGF therapy are also available [112]. Second-line treatments include classical drugs, such as docetaxel, irinotecan, as well as newer antibodies against tyrosine kinase receptors (TKRs) (trastuzumab and cetuximab), and some TKIs (erlotinib and gefitinib) [113]. The activity of several ABC proteins reduces the sensitivity to many of these drugs (Table 5).

### 5.1. MDR1 in Gastric Adenocarcinoma

The relevance of MDR1 in GAC chemoresistance is unclear. MDR1 is barely detectable in normal gastric mucosa, either by mRNA expression analysis [125] or immunohistochemistry [126]. Some studies have reported enhanced MDR1 expression in the early stages of GAC development, associating such expression with a poor prognosis due to increased resistance to chemotherapy [127,128]. However, other authors have found intracellular localization of the protein in GAC-derived cells, which precludes any role of the pump as a drug exporter [129]. Elevated MDR1 expression has been observed in GACs from patients classified as poor responders to platinum-based therapy alone or in combination with epirubicin [114,115]. This was surprising because the cytotoxic activities of these drugs were not expected to be affected by MDR1 expression levels [130]. Nevertheless, studies carried out using in vitro cellular models have demonstrated the involvement of MDR1 in GAC resistance to chemotherapeutic treatment with oxaliplatin [116], cisplatin [117], and epirubicin [118]. This points to MDR1 as a potential target for gene therapy to improve the response to the pharmacological treatment of patients with advanced GAC.

### 5.2. MRP1 in Gastric Adenocarcinoma

MRP1 is highly expressed in gastric mucosa and GAC [131,132]. The expression levels of this protein have been extensively investigated in several studies, which have reported elevated MRP1 expression in a variable proportion of cases ranging from 12 to 89% of GACs analyzed [132,133,134]. In most GAC specimens, MRP1 expression levels do not differ substantially from those determined in adjacent healthy tissue, although decreased MRP1 expression has been reported in a small proportion of samples analyzed [133]. The study of GAC-derived cell lines reveals a great variability in MRP1 expression, which inversely correlates with the sensitivity of these cells to anticancer drugs, such as etoposide, vincristine, epirubicin, doxorubicin, and vinblastine [135]. MRP1 has been proposed as a marker of chemoresistance in GAC, mainly associated with acquired resistance to cisplatin, although this drug is not believed to be a substrate of MRP1. Following treatment with anticancer platinum-based regimens, increased chemoresistance associated with higher MRP1 expression has been observed in both GAC patients and the cisplatin-resistant GAC-derived cell line KATOIII/DDP [119]. In addition, recent studies suggest that long non-coding RNAs (lncRNAs) are involved in the acquisition of the MDR phenotype, a process in which increased MRP1 expression is involved [136,137].

### 5.3. MRP2 and MRP3 in Gastric Adenocarcinoma

The relevance of MRP2 and MRP3 in developing the MDR phenotype in GAC cells is likely minor and null, respectively. MRP2 expression is inversely correlated with differentiation, being higher in poorly differentiated gastric tumors [138]. In GAC-derived cell lines, MRP2 expression varies from low to moderate [139,140].

Besides, MRP3 has not been detected at the protein level in healthy stomachs and GAC [44,132]. The same occurs in GAC-derived cells, except for SNU601 cells with acquired resistance to cisplatin, in which an increased MRP3 expression has been found [141].

### 5.4. MRP4 and MRP5 in Gastric Adenocarcinoma

MRP4 is abundantly expressed in healthy gastric mucosa and GAC [122,132]. In GAC biopsies, the MRP4 expression was significantly higher than in normal gastric tissue [142]. Furthermore, MRP4 levels are upregulated in several gastric cell lines, especially in those that have developed chemoresistance [120]. The inhibition of MRP4 expression in these cells by siRNA increases the sensitivity to 5-FU through cell cycle arrest and activation of apoptosis via BCL2/BAX [120]. Similarly, increased MRP4 expression has been described in the cisplatin-resistant cell line SGC7901, whose sensitivity to this drug was enhanced after *ABCC4* silencing using siRNA [121]. MRP4 has also been associated with reduced GAC response to dasatinib [122].

Scarce information on MRP5 expression in GAC is available. Although MRP5 is known to transport 5-FU, there is no data on the relationship between this pump and the response of GAC to 5-FU.

### 5.5. BCRP in Gastric Adenocarcinoma

Immunohistochemical analysis of BCRP shows homogeneous and intense staining in the plasma membrane and intracellular compartment of untreated GAC [143]. In addition, in vitro studies have revealed increased *ABCG2* mRNA levels in GAC cells exposed to cisplatin [144]. In GAC patients, elevated BCRP levels in tumor samples obtained before chemotherapy were associated with shorter OS [123]. Furthermore, after treatment with cisplatin and 5-FU, the residual cells have elevated expression of hedgehog (Hg) target genes GLI1 and GLI2, suggesting activation of Hg signaling. This pathway is a crucial regulator for putative cancer stem cells. Thus, enhanced GLI1/GLI2 expression is accompanied by BCRP upregulation, which is associated with decreased OS and increased risk of cancer relapse [124]. In addition, GLI2 knockdown sensitized GAC cells to 5-FU treatment. Because of the possible role of BCRP in GAC chemoresistance, identifying novel BCRP inhibitors able to enhance the response to anticancer drugs transported by this pump could be helpful. For example, genistein-induced inhibition of BCRP expression in GAC-derived cells is associated with increased sensitivity to 5-FU and cisplatin [145]. Furthermore, using ribozymes in GAC cells to reduce BCRP mRNA increased sensitivity to BCRP substrates [146].

## 6. Pancreatic Ductal Adenocarcinoma

Pancreatic ductal adenocarcinoma (PDAC) is another aggressive malignancy affecting the digestive system. The number of deaths caused by this cancer per year worldwide is close to 470,000, i.e., 4.7% of deaths due to cancer. The standard-of-care therapy for PDAC patients consists of 5-FU, leucovorin, irinotecan, and oxaliplatin (FOLFIRINOX) [147], although some patients receive gemcitabine alone or combined with nab-paclitaxel or erlotinib. The benefit of chemotherapy is minimal because PDAC is characterized by its high chemoresistance, due in part to the elevated activity of ABC pumps accounting for the reduction of intracellular concentration of these drugs [148] (Table 6).

### 6.1. MDR1 in Pancreatic Ductal Adenocarcinoma

As in other epithelial cells, MDR1 is expressed in the apical membrane of epithelial cells lining the pancreatic ducts [23], where it participates in the transport of components of the pancreatic secretion. Immunohistochemical analysis revealed that MDR1 expression is higher in non-treated PDAC than in healthy ducts [162,163]. Intense positive staining was observed in 52 out of 71 PDAC analyzed, and this was associated with a better prognosis in patients who did not receive chemotherapy [163]. MDR1 expression in PDAC-derived cell lines is a heterogeneous feature, ranging from undetectable or low to high [149,164,165]. The abundance of *ABCB1* mRNA has been associated with resistance to gemcitabine [149] and taxanes, but not to 5-FU [166]. Gemcitabine-resistant pancreatic cell lines established by dose-escalation of the drug showed stemness characteristics and MDR1 overexpression [167].

### 6.2. MRP1 in Pancreatic Ductal Adenocarcinoma

MRP1 is expressed in fibroblasts but not in pancreatic acinar/ductal cells and PDAC [168]. Although MRP1 mRNA expression was high in 32 PDAC specimens compared to adjacent non-tumor tissue, minimal contribution to the poor response to treatment was attributed to this transporter [169]. However, more recent studies have demonstrated that MRP1 overexpression is associated with 5-FU [151] and gemcitabine resistance [152,170] in PDAC cells in vitro. Interestingly, molecular communication between cancer-associated fibroblasts (CAFs) and PDAC cells has been suggested as the mechanism triggering MRP1 downregulation and, consequently, reduced gemcitabine resistance in PDAC cells [153].

### 6.3. MRP2 in Pancreatic Ductal Adenocarcinoma

Immunohistochemical analyses have supported that MRP2 is not expressed in healthy exocrine pancreatic tissue [138,168]. However, there is controversy regarding the expression of this pump in PDAC, because although it was not detected by some groups [168], other studies have detected its presence in 91% of 67 tumor samples from patients who had not previously received any treatment [161]. Different research detected intracellular and plasma membrane staining of MRP2 in 77.5% of 40 tumor samples analyzed [171]. Patients bearing the MRP2 variant Gly40Ala showed a weak association with survival and tumor response to gemcitabine therapy and, surprisingly, this association diminished in patients receiving gemcitabine/cisplatin plus radiotherapy [154]. Regarding human pancreatic cell lines, one study described a low abundance of MRP2 mRNA in some PDAC-derived cells, but protein expression by Western blot was not found in any of the seven cell lines tested [156]. Another study reported moderate MRP2 mRNA expression in five cell lines and induction of its expression in cisplatin-resistant cells [171]. Besides, indirect downregulation of MRP2 gene expression was suggested to induce gemcitabine sensitivity in PDAC cell lines [155].

### 6.4. MRP3 in Pancreatic Ductal Adenocarcinoma

MRP3 is expressed in the basolateral membrane of normal pancreatic acinar/ductal cells and PDAC, with higher mRNA levels in more differentiated tumors [168]. Increased expression of MRP3 was associated with lower survival of PDAC patients and it was suggested to play an essential role in tumor growth in vivo [157]. MRP3 expression is variable among PDAC-derived cell lines and was upregulated both at mRNA and protein levels [156,157] in an established 5-FU resistant cell line [156], but not in gemcitabine-resistant cells [160].

### 6.5. MRP4 in Pancreatic Ductal Adenocarcinoma

MRP4 is localized in the plasma membrane of both ductal and acinar pancreatic cells and PDAC cells [168,172]. Still, there is controversy regarding the expression of this export pump in PDCA compared with normal ductal cells. Thus, one study reported similar expression in PDAC and healthy tissue, but another found higher expression in PDAC [168,172]. Variable mRNA and protein MRP4 levels in seven PDAC-derived cell lines have been reported. Upregulation of MRP4 in response to increased intracellular cAMP levels [173] and in 5-FU resistant cells [156] has been described.

### 6.6. MRP5 and MRP6 in Pancreatic Ductal Adenocarcinoma

MRP5 is also localized in the basolateral membrane of the duct and acinar cells and the plasma membrane of PDAC cells [168]. MRP5 mRNA levels were higher in PDAC than in normal tissue and were not associated with tumor grade or stage [168]. Several studies using PDAC-derived cell lines have shown that *ABCC5* mRNA levels in these cells correlated significantly with their refractoriness to 5-FU [158]. Exposure of PDAC cells to gemcitabine or 5-FU induced MRP5 upregulation, which was associated with enhanced resistance to these anticancer agents [169,171,174].

The information regarding MRP6 expression in PDAC is scarce. The abundance of *ABCC6* mRNA in healthy pancreas and PDAC specimens obtained before chemotherapy was very low [168].

### 6.7. BCRP in Pancreatic Ductal Adenocarcinoma

The abundance of *ABCG2* mRNA in healthy pancreatic tissue is low. In contrast, although with marked interindividual variability, considerable *ABCG2* mRNA levels have been found in PDAC samples from 31 patients undergoing partial pancreatectomy [168]. Heterogeneity in BCRP expression was confirmed by immunohistochemistry, which revealed positive staining in 73% of 65 PDAC samples analyzed, as well as an association between high BCRP expression, early recurrence, and poor survival of patients treated with adjuvant gemcitabine-based chemotherapy [161].

## 7. Colorectal Carcinoma

The available drug therapy to treat colorectal carcinoma (CRC) provides limited beneficial effects in patients with advanced disease [175]. Thus, close to 940,000 people die from CRC each year, which represents 9.4% of deaths due to cancer. Both conventional chemotherapy, based mainly on 5-FU and other pyrimidine analogs (e.g., capecitabine, trifluridine, and tipiracil), platinum agents (e.g., oxaliplatin), and irinotecan, and targeted therapy, based on TKIs (e.g., regorafenib), and monoclonal antibodies against TKRs (e.g., aflibercept, bevacizumab, cetuximab, panitumumab, and ramucirumab) are used [175]. Immunotherapy has recently been approved to treat CRC. The role of ABC proteins in CRC chemoresistance is commented below (Table 7).

### 7.1. MDR1 in Colorectal Carcinoma

MDR1 is highly expressed in the colon mucosa [23]. The abundance of *ABCB1* mRNA is increased in most CRCs, being significantly higher in well-differentiated than in poorly differentiated tumors [187] regardless of tumor location and size [188]. Immunohistochemical analysis of CRC biopsies also revealed a high proportion of MDR1 positivity among analyzed tumors [188]. Nevertheless, MDR1 is believed to play a minor role in CRC resistance to pharmacological treatment based on 5-FU, platinum derivatives, irinotecan, and its active metabolites because these drugs are not transported by MDR1 [189,190]. However, this pump contributes to the CRC intrinsic resistance to anthracyclines, *Vinca* alkaloids, epipodophyllotoxins, and taxanes, all of which are MDR1 substrates [191]. Unlike in tumors, in CRC-derived cell lines, MDR1 expression is higher in poorly differentiated than in more differentiated ones. Moreover, their MDR1 expression levels parallel the resistance to its drug substrates, such as anthracyclines and vincristine [174].

### 7.2. MRP1 in Colorectal Carcinoma

The role of MRP1 in CRC chemoresistance is controversial. Several studies have reported the lack of association [190,192], whereas others have observed a link between TNM staging and differentiation grade and enhanced MRP1 expression in CRC biopsies, revealed by positive immunohistochemical staining [193]. Moreover, in vitro testing has demonstrated that MRP1 is associated with resistance to oxaliplatin, 5-FU, and bevacizumab [177,178,194]. Besides, the detection of MRP1 in circulating tumor cells in patients with CRC has recently been suggested as a biomarker of irinotecan resistance [179,195]. Furthermore, the presence of the intron variants *ABCC1* rs17501011 (c.49-20550G>A), *CES1* rs9921399 (c.52+538A>G), *UGT1A* rs1113193 (c.855+41929G>A for *UGT1A8* and c.855+23114G>A for *UGT1A10*) was related to lower OS in FOLFIRI-treated CRC patients [180].

### 7.3. MRP2 in Colorectal Carcinoma

High levels of MRP2 mRNA have been found in CRC compared with tissue from non-tumor surrounding mucosa and healthy individuals. Elevated MRP2 expression is considered one of the most critical mechanisms of chemoresistance, leading to the failure of CRC treatment based on cisplatin or oxaliplatin [181,196]. In several CRC-derived cell lines, MRP2 is constitutively expressed, their sensitivity to cisplatin being dependent on their *ABCC2* mRNA levels [182], which correlate with reduced intracellular cisplatin accumulation [197]. Long-term exposure of these cells to cisplatin results in a marked stimulation of the expression of several ABC proteins, particularly MRP2 [198,199]. In addition, when cisplatin-sensitive cells were stably transfected with *ABCC2* cDNA, they acquired drug resistance [77], which was reversed by incubation with probenecid, an MRP2 inhibitor [197].

### 7.4. MRP3 in Colorectal Carcinoma

The role of MRP3 in the response of CRC to chemotherapy seems irrelevant. Indeed, in surgically resected CRC, no relationship between MRP3 expression and the sensitivity to anticancer agents, such as doxorubicin, mitomycin C, cisplatin, 5-FU, etoposide, and camptothecin derivatives, has been found [181]. Moreover, MRP3 mRNA levels in CRC and colorectal polyps compared with non-tumor tissues are decreased or unchanged [181,200]. However, upregulation of MRP3 in CRC-derived cell lines contributed to resistance to oxaliplatin [183] and etoposide [184]. In addition, short-term exposure in vitro to cisplatin [199] or oxaliplatin and 5-FU [201] results in enhanced MRP3 expression.

### 7.5. MRP4 in Colorectal Carcinoma

Compared with healthy tissue, MRP4 expression is higher in human CRC, colorectal polyps, and CRC cell lines [202]. Despite the ability of MRP4 to transport 5-FU and irinotecan, there is no strong evidence linking MRP4 expression to the lack of response of CRC patients to these drugs. The rs3742106 variant of *ABCC4* (c.*38A>C/T) affects the binding of miR-3190-5p to *ABCC4* 3′-UTR, which reduces its expression [185]. Thus, in patients with enhanced expression of this MRP4 variant, 5-FU concentrations in tumor cells are reduced [185]. It should be considered that CRC is a tumor with a marked inflammatory microenvironment. In this context, MRP4 plays a role in regulating the levels of prostaglandin E2 (PGE2), the most abundant COX-2-derived pro-carcinogenic prostaglandin in the CRC microenvironment [203]. Some authors have investigated the effect of celecoxib, a COX-2 inhibitor, in combination with irinotecan and oxaliplatin against CRC. However, trials have not shown an advantageous effect of this combination, which may be due to the fact that celecoxib induces MRP4 upregulation [204].

### 7.6. MRP5 in Colorectal Carcinoma

Genetic variants of *ABCC5* (along with those of the *ABCG1* and *SLCO1B1*) have been associated with the gastrointestinal toxicity of its substrate irinotecan [205]. Its administration should be avoided in combination with celecoxib because this drug increases MRP5 expression [204]. Experiments with cells transfected with MRP5 show increased efflux of 5-FU, adefovir, and purine analogs [104,206]. These studies also demonstrated that, in addition to 5-FU, MRP5 also confers resistance in CRC to several anticancer agents, including methotrexate, pemetrexed, doxorubicin, and the platinum-containing drugs cisplatin and oxaliplatin [104]. However, the role of MRP5 expression in CRC chemoresistance in the clinical setting has not been established.

### 7.7. BCRP in Colorectal Carcinoma

Compared with paired non-tumor tissue, a significantly lower abundance of *ABCG2* mRNA was determined in CRC biopsies obtained before the patients received antitumor therapy [198]. However, BCRP is highly expressed in CD133-positive cells from human CRC. These are thought to be putative cancer stem-like cells with an associated lack of response, leading to tumor recurrence and metastasis [186,207]. High BCRP levels have been found in CRC-derived mitoxantrone- and cisplatin-resistant cell lines [199,208]. Downregulation of this efflux pump significantly enhances the efficacy of 5-FU and oxaliplatin in vitro and in vivo [186].

### 7.8. Other ABC Pumps in Colorectal Carcinoma

Regarding the role of other ABCs in the MDR phenotype of CRC, *ABCB5* has been proposed as a marker of refractoriness to 5-FU treatment in these patients. This was due to the high levels of expression of this protein that was found in patients who did not respond to 5-FU-based chemotherapy regimens. Interestingly, the relationship between *ABCB5* overexpression and 5-FU resistance was confirmed in a CRC xenograft model that had undergone 5-FU monotherapy [176].

## 8. Conclusions

Although there is still some confusion about the actual relevance of ABC pumps in the response of hepatobiliary, pancreatic, and gastrointestinal cancers to pharmacological treatment, there is a clear consensus on the need to consider them in future strategies to improve the efficacy of classical and newer drugs. The first step in this direction is to correctly identify the members of this superfamily of proteins expressed in each type of cancer (Figure 1). However, the existence of interindividual variability makes it necessary to define the MOC pattern on a tumor-by-tumor basis, even in a dynamic way, because it can vary during tumor progression and in response to chemotherapy. One important conclusion of this descriptive review is that each type of cancer has its own major characteristics defining its ABC expression pattern, which should be refined by deeper individual analyses and considered before starting any pharmacological treatment to get the highest probability of success in each patient. This information would also be required to develop novel strategies for sensitization.

## Figures and Tables

**Table 1:** ABC proteins involved in the transport of common antitumor drugs used in the treatment of hepatobiliary, pancreatic, and gastrointestinal tumors.

Pharmacological Groups	Drugs	Cancers	ABC Pumps		
Alkylating Drugs	Cyclophosphamide	HB		MRP1, 4	
Anthracyclines	Doxorubicin	GAC, HB, HCC	MDR1	MRP1, 2, 6	BCRP
Camptothecins	Irinotecan	BTC, CRC, GAC, HB, PDAC	MDR1	MRP1, 2, 4, 6	BCRP
TKIs	Sorafenib	HCC	MDR1	MRP1, 2, 3	BCRP
	Regorafenib	CRC, HCC	MDR1	MRP2	
	Lenvatinib	HCC	MDR1	MRP2	
	Cabozantinib	HCC	MDR1	MRP2	
Platinum Derivatives	Cisplatin	BTC, GAC, HB, HCC		MRP2, 6	
	Oxaliplatin	BTC, CRC, GAC, PDAC		MRP2, 6	
Podophyllotoxins	Etoposide	BTC, HB	MDR1	MRP1, 2	
Pyrimidines	5-FU	BTC, CRC, GAC, HB, HCC, PDAC		MRP1, 4, 5	BCRP
	Gemcitabine	BTC, PDAC	MDR1	MRP2, 4, 5, 6	
Taxanes	Docetaxel	GAC	MDR1	MRP1	
Vinca Alkaloids	Vinblastine	HB	MDR1	MRP1, 2	

5-FU, 5-Fluorouracil; BCRP, breast cancer resistance protein; BTC, biliary tract cancer; CRC, colorectal carcinoma; GAC, gastric adenocarcinoma; HB, hepatoblastoma; HCC, hepatocellular carcinoma; MDR, multidrug resistance protein; MRP, multidrug resistant-associated protein; PDCA, pancreatic ductal adenocarcinoma; TKI, tyrosine kinase inhibitor.

**Table 2:** Role of ABC proteins in the chemoresistance of hepatocellular carcinoma (HCC).

Change	Drugs Affected	Impact	Ref.
MDR1 upregulation	Sorafenib	Reduced OS	[8,9]
	Doxorubicin	Worse prognosis	[10]
MDR1 variant rs1045642	Sorafenib	Better clinical evolution	[11]
ABCB5 upregulation	Doxorubicin	Decreased cell sensitivity in vitro	[12]
MRP1 upregulation	Sorafenib	Worse response	[13]
MRP2 upregulation	Sorafenib	Decreased cell sensitivity in vitro	[14]
MRP3 upregulation	Sorafenib	Decreased cell sensitivity in vitro	[15]
MRP4 upregulation	Cisplatin	Decreased cell sensitivity in vitro	[16]
MRP5 upregulation	5-FU, cisplatin, doxorubicin	Decreased cell sensitivity in vitro	[17,18,19]
BCRP upregulation	Doxorubicin	Decreased cell sensitivity in vitro	[20]
	Sorafenib	Worse response	[9,21]
BCRP variants rs2231137 and rs2231142	Sorafenib	Better clinical evolution	[11]
ABCF1 upregulation	Cisplatin, doxorubicin	Decreased cell sensitivity in vitro and in vivo	[22]

5-FU, 5-Fluorouracil; BCRP, breast cancer resistance protein; MDR, multidrug resistance protein; MRP, multidrug resistant-associated protein; OS, overall survival.

**Table 3:** Role of ABC proteins in chemoresistance of biliary tract cancer (BTC).

Change	Drugs Affected	Impact	Ref.
MDR1 upregulation	Gemcitabine	Reduced response	[60]
MRP1 upregulation	5-FU, Gemcitabine	Decreased cell sensitivity in vitro	[61,62]
	Cisplatin, Gemcitabine	Worse prognosis	[63,64]
MRP3 downregulation	5-FU	Increased cell sensitivity in vitro	[65]
MRP5 downregulation	Gemcitabine	Increased cell sensitivity in vitro	[66]
MRP6 downregulation	Gemcitabine	Increased cell sensitivity in vitro	[66]
MDR1, MRP3, and BCRP upregulation	5-FU	Reduced response	[67]

5-FU, 5-Fluorouracil; BCRP, breast cancer resistance protein; MDR, multidrug resistance protein; MRP, multidrug resistant-associated protein.

**Table 4:** Role of ABC proteins in chemoresistance of hepatoblastoma (HB).

Protein Change	Drugs Affected	Impact	Ref.
MDR1 upregulation	Doxorubicin	Decreased cell sensitivity in vitro	[89]
MRP1 upregulation	Doxorubicin	Reduced response	[33]
MRP2 upregulation	Cisplatin	Reduced response	[90]

MDR, multidrug resistance protein; MRP, multidrug resistant-associated protein.

**Table 5:** Role of ABC proteins in chemoresistance of gastric adenocarcinoma (GAC).

Change	Drugs Affected	Impact	Ref.
MDR1 upregulation	Cisplatin, epirubicin	Worse response	[114,115]
	Cisplatin, oxaliplatin, epirubicin	Decreased cell sensitivity in vitro	[116,117,118]
MRP1 upregulation	Cisplatin	Worse response	[119]
MRP4 downregulation	5-FU	Increased cell sensitivity in vitro	[120]
	Cisplatin	Increased cell sensitivity in vitro	[121]
MRP4 upregulation	Dasatinib	Decreased cell sensitivity in vitro and in vivo	[122]
BCRP upregulation	Cisplatin, 5-FU	Reduced OS	[123,124]

5-FU, 5-Fluorouracil; BCRP, breast cancer resistance protein; MDR, multidrug resistance protein; MRP, multidrug resistant-associated protein; OS, overall survival.

**Table 6:** Role of ABC proteins in chemoresistance of pancreatic ductal adenocarcinoma (PDAC).

Change	Drugs Affected	Impact	Ref.
MDR1 upregulation	Gemcitabine	Decreased cell sensitivity in vitro	[149,150]
MRP1 upregulation	5-FU	Decreased cell sensitivity in vitro	[151,152]
MRP1 downregulation	Gemcitabine	Increased cell sensitivity in vitro	[153]
MRP2 upregulation	Gemcitabine	Reduced OS	[154]
MRP2 downregulation	Gemcitabine	Increased cell sensitivity in vitro	[79,155]
MRP3 upregulation	5-FU	Decreased cell sensitivity in vitro	[156,157]
MRP4 upregulation	5-FU	Decreased cell sensitivity in vitro	[156]
MRP5 upregulation	5-FU	Decreased cell sensitivity in vitro	[158]
	Gemcitabine	Decreased cell sensitivity in vitro	[159,160]
BCRP upregulation	Gemcitabine	Worse prognosis	[161]

5-FU, 5-Fluorouracil; BCRP, breast cancer resistance protein; MDR, multidrug resistance protein; MRP, multidrug resistant-associated protein; OS, overall survival.

**Table 7:** Role of ABC proteins in chemoresistance of colorectal carcinoma (CRC).

Change	Drugs Affected	Impact	Ref.
MDR1 upregulation	Doxorubicin, vincristine	Decreased cell sensitivity in vitro	[174]
ABCB5 upregulation	5-FU	Worse response	[176]
MRP1 upregulation	5-FU, oxaliplatin	Decreased cell sensitivity in vitro	[177,178]
	Irinotecan	Worse response	[179]
MRP1 variant rs17501011	5-FU, irinotecan	Reduced OS	[180]
MRP2 upregulation	Cisplatin	Worse response	[181]
	Cisplatin	Decreased cell sensitivity in vitro	[182]
MRP3 upregulation	Etoposide, oxaliplatin	Decreased cell sensitivity in vitro	[183,184]
MRP4 variant rs3742106	5-FU	Worse response	[185]
MRP5 upregulation	5-FU, doxorubicin, cisplatin, oxaliplatin	Decreased cell sensitivity in vitro	[104]
BCRP downregulation	5-FU, oxaliplatin	Increased cell sensitivity in vitro	[186]

5-FU, 5-Fluorouracil; BCRP, breast cancer resistance protein; MDR, multidrug resistance protein; MRP, multidrug resistant-associated protein; OS, overall survival.

## Abbreviations

5-FU, 5-fluorouracil; BCRP, breast cancer resistance protein; BTC, biliary tract cancer; CAF, cancer-associated fibroblast; CCA, cholangiocarcinoma; CRC, colorectal carcinoma; eCCA, extrahepatic CCA; GAC, gastric adenocarcinoma; GBC, gallbladder cancer; HB, hepatoblastoma; HCC, hepatocellular carcinoma; iCCA, intrahepatic CCA; LCSC, liver cancer stem cells; MDR, multidrug resistance protein; MRP, multidrug resistant-associated protein; OS, overall survival; PDCA, pancreatic ductal adenocarcinoma; TACE, transarterial chemoembolization; TKI, tyrosine kinase inhibitor; TKR, tyrosine kinase receptor.
